# Chemical Composition, Antioxidant, Antimicrobial, Antidiabetic, and Butyrylcholinesterase Inhibitory Activities in Vitro of the Essential Oil and Crude Extracts of the Aerial Parts of *Thymus ciliatus*

**DOI:** 10.1002/cbdv.202403318

**Published:** 2025-04-22

**Authors:** Nadia Djermane, Mostapha Brahmi, Djallal Eddine Adli, Micaela Gliozzi, Vincenzo Musolino, Rebbas Khellaf, Ramazan Erenler, Rabah Arhab, Stefania Garzoli

**Affiliations:** ^1^ Department of Natural and Life Sciences Faculty of Exact Sciences and Natural and Life Sciences, Laboratory of Bioactive Molecules and Applications Larbi Tebessi University Tebessa Algeria; ^2^ Department of Natural and Life Sciences Faculty of Natural and Life Sciences, Larbi Tebessi University Tebessa Algeria; ^3^ Department of Biology Faculty of Sciences and Technology, University of Relizane Relizane Algeria; ^4^ Department of Biology, Faculty of Sciences, Laboratory of Biotoxicology, Pharmacognosy and Biological Valorization of Plants (LBPVBP) University of Dr Moulay Tahar Saida Algeria; ^5^ Department of Health Sciences Laboratory of Pharmaceutical Biology IRC‐FSH Center University “Magna Græcia” of Catanzaro Catanzaro Italy; ^6^ Laboratory of Agro‐Biotechnology and Nutrition in Arid and Semi‐Arid Areas Ibn Khaldoun University Tiaret Algeria; ^7^ Department of Chemistry Faculty Of Art and Science, Plant Research Laboratory Tokat Gaziosmanpasa University Tokat Turkey; ^8^ Department of Chemistry and Technologies of Drug Sapienza University Rome Italy

**Keywords:** antioxidant activity, antimicrobial activity, butyrylcholinesterase activity, GC‐MS, α‐glucosidase

## Abstract

This study explores the potential of *Thymus ciliatus* as a natural source of bioactive compounds by investigating its chemical composition and in vitro biological activities, including antioxidant, antimicrobial, antidiabetic, and anti‐Alzheimer properties. The analysis of the essential oil was performed using gas chromatography‐mass spectrometry revealing α‐terpinenyl acetate (18.74%) and camphor (10.62%) as the major components. Antioxidant activity was evaluated using six methods. Antimicrobial activity was assessed using disc diffusion and well diffusion techniques. Antidiabetic activity was measured through a colorimetric assay, while anti‐Alzheimer activity was evaluated against butyrylcholinesterase. The results demonstrated that extracts from polar and medium‐polar solvents exhibited the highest antioxidant activity, followed by low‐polar solvent extracts. The essential oil of *T. ciliatus* displayed significant antimicrobial activity, particularly against *Staphylococcus aureus*, *Bacillus cereus*, and *Candida albicans*. Crude extracts also showed antimicrobial activity across all tested strains. The aqueous extract exhibited the strongest antidiabetic activity against α‐glucosidase (half‐maximal inhibitory concentration [IC_50_] = 2.56 ± 0.06 µg/mL), followed by the essential oil (IC_50_ = 57.11 ± 4.39 µg/mL). Furthermore, the dichloromethane extract demonstrated the highest anti‐Alzheimer activity with an IC_50_ of 0.26 ± 0.20 µg/mL. Based on these results, *T. ciliatus* represents a promising source of bioactive substances with potential therapeutic applications.

## Graphical abstract

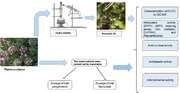

## 1 Introduction

The genus Thymus (thyme) is one of the most important genera in a number of species of the family Lamiaceae and includes about 220 species of aromatic perennial herbs and subshrubs [1]. This genus is distributed over the Old World and the coast of Greenland, the species is found in the Macaronesian region, North Africa, the Sinai Peninsula, and across western and eastern Asia. Its primary distribution, however, lies within the Mediterranean basin [2]. Plants of the Thymus genus are among the world's most renowned species, frequently consumed as herbal teas., and flavoring agents (condiment and spice) [3]. In addition, *Thymus* species are known to be used in traditional medicine as remedies for various diseases, for example, pulmonary infection, toux, grippe, bronchitis, and some gastrointestinal disorders [4]. These species have also been used as a carminative, diuretic, urinary disinfectant, and vermifuge [2, 5]. In fact, many studies have shown that some species of *Thymus* have strong antioxidant and antimicrobial activity [6]. In parallel to their biological properties, natural extracts have other applications, such as anti‐inflammatory, immunomodulatory, spasmolytic, and sedative effects [7], thus these biological and pharmacological virtues have been mainly due to the richness of essential oils contained in the majority of *Thymus* species, as well as to non‐volatile compounds such as polyphenolics and flavonoids [8]. Essential oils are mainly composed of monoterpene hydrocarbons, oxygenated monoterpenes, and sesquiterpenes [9]. The two main phenolic monoterpenes, namely thymol, and carvacrol, have been considered, along with other volatile compounds, as antiseptic agents in several pharmaceutical fields and as flavoring agents in many food products [9, 10]. The genus Thymus (Lamiaceae) is broadly distributed, comprising eight sections and approximately 220 species predominantly found throughout the Mediterranean basin [4]. Within the Algerian flora, the genus Thymus from this family includes 12 species, nine of which are endemic [11]. Among them is *Thymus ciliatus* (Desf.) Benth is an endemic species of North Africa. This species includes three subspecies: ssp. *euciliatus* Maire, ssp. *coloratus* (Boiss. et Reut.) Batt. and ssp. *munbyanus* (Boiss. et Reut.) Batt [12]. Indeed, in North African folk medicine, Thymus plants are used as powders, decoctions, or infusions to treat various diseases such as toux, fever, and diarrhea [13].

The present research was aimed at characterizing the chemical composition of the essential oil extracted from the aerial part of *T. ciliatus* (Desf), originating from eastern Algeria (Djbel Taref, wilaya of Oum El Bouaghi) Algeria. In addition, some biological activities, such as antioxidant, antimicrobial, antidiabetic, and butyrylcholinesterase (BChE) inhibitory activity of the essential oil and crude extracts of the species were studied to evaluate the possibility in the pharmacological field.

## 2 Results and Discussion

### 2.1 Extraction Yield of Essential Oil and Crude Extracts of *T. ciliatus*

The essential oil extracted by hydro‐distillation from the aerial parts of our plant *T. ciliatus* yielded 0.23% (Table 1). Our results are in disagreement with the studies of Bousmaha‐Marroki et al. [14], who obtained a yield between (3.0 and 5.1%) in the essential oil of *T. ciliatus* collected in different regions of Tlemcen (Algeria). Another research conducted by Giordani et al. [15] on different thyme species including the species *T. ciliatus* native to Jebel Ansel in Guelma (Algeria), reported a yield of (2% and 3%). In the same context, Amarti et al. [16] reported that the EO yield for the aerial part of the same species native to Morocco was about (1.2%). These variations in essential oil yield depend on several factors such as the time of harvest (vegetative, flowering, or post‐flowering), the different organs of the plant [17], the nature and geographical origin of the plant [18], and the extraction technique [19]. On the other side, the yield of crude extracts (aqueous, methanolic, acetonic, and dichloromethane) of *T. ciliatus* is respectively 3.4%, 8.95%, 3.9%, and 2.7% (Table 1), from these results it appears that the highest yield is that of the methanolic extract; this is in agreement with the studies of Sonia et al. [20], who demonstrated that alcohols have the ability to increase the permeability of cell walls facilitating the extraction of a greater number of polar, medium and low polarity molecules. The different organic extracts of our plant contain decreasing yields: methanolic extract>acetone extract>dichloromethane extract, which means that the content of the extract varies according to the polarity of the extraction solvent used but also the extraction technique plays an important role in the yield rate of the extract.

**TABLE 1:** Yields of essential oil and various crude extracts of *T. ciliatus*.

Crude extracts and essential oil	Yield of *T. ciliatus*
Aqueous	3.4%
Methanolic	8.95%
Acetonic	3.9%
Dichloromethane	2.7%
Essential oil	0.23%

### 2.2 Chemical Composition of the Essential Oil of *T. ciliatus*

**FIGURE 1:**
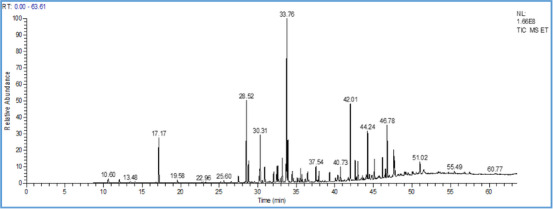
Chromatogram of the essential oil of *T. ciliatus*.

The chemical characterization by gas chromatography‐mass spectrometry (GC‐MS) analysis allowed us to identify 20 compounds representing 95.26% of the total. The majority of compounds are α‐terpinenyl acetate (18.74%), camphor (10.62%), caryophyllene oxide (9.58%), trans‐nerolidyl acetate (6.63%), 1,8‐cineole (6.55%), epiglobulol (5.59%), bornyl acetate (5.56%), and borneol (5.15%) (Figure 1 and Table 2). However, previous studies have focused on the variation in the chemical composition of essential oils of the species *T. ciliatus* from different regions of Algeria. Indeed, 28 components were identified for the essential oil of *T. ciliatus* flowers (unspecified subspecies) from the region of Ain M'Lilan (East of Algeria), with thymol (54.98%), γ‐terpinene (11.33%), p‐cymene (6.66%), and carvacrol (4.96%) as the major components [21]. In addition, the chemical composition of 8 samples of the essential oil from the aerial part of *T. ciliatus* ssp. *euciliatus* collected in different regions of Tlemcen (Western Algeria), was described by Bousmaha et al. [14].

**TABLE 2:** Chemical composition of the essential oil of the species *T. ciliatus*.

No	RT	Compounds	^a^RI	^b^RI	%
1	17.2	1,8‐cineole	1061	1059	6.5
2	28.52	Linalool	1083	1082	2.6
3	28.82	α‐Fenchyl alcohol	1123	1121	2.1
4	30.31	trans‐Pinocarveol	1140	1138	2.1
5	30.89	cis‐Verbenol	1144	1142	2.9
6	32.46	Camphor	1153	1151	10.6
7	32.59	Borneol	1167	1165	5.1
8	33.17	Terpinen‐4‐ol	1182	1179	2.4
9	33.66	trans‐Carveol	1219	1217	1.9
10	33.76	Bornyl acetate	1255	1252	5.6
11	33.9	α‐Terpinenyl acetate	1349	1346	18.7
12	37.54	Aromadendrene	1441	1439	2.1
13	42.01	Epiglobulol	1536	1532	5.6
14	42.97	Caryophyllene oxide	1574	1573	9.6
15	44.24	Spathulenol	1577	1575	2.4
16	45.12	Viridiflorol	1592	1590	2.5
17	46.17	α‐Cadinol	1653	1652	3.3
18	46.78	β‐Eudesmol	1655	1654	2.5
20	47.7	trans‐nerolidyl acetate	1683	1680	6.6
**TOT**.			95.3		

^a^ Retention index calculated from retention times relative to that of *n*‐alkane series, ^b^Retention index of reference, %: Abundance percentage.

The quantity and nature of the dominant constituents vary considerably from one sample to another depending on the region of harvest of the plant: carvacrol (72.4%–80.3%), p‐cymene (4.2%–7.2%), γ‐terpinene (1.6%–7.8%), and β‐caryophyllene (1.7%–2.2%) accompanied by oxide (0.2%–0.8%) and dihydroabietane (tr–0.3%). In contrast, the essential oil of *T. ciliatus* from Jebel Ansel (East of Algeria) is dominated by thymol (60.52%) [15]. Therefore, the chemical composition of essential oils of *T. ciliatus* from different regions of Algeria presents a great diversity in terms of the majority of compounds.

The same for *T. ciliatus* from Morocco in fact, Amarti et al. [16], showed that the essential oil of the aerial part of *T. ciliatus* harvested in the region of Azrou is characterized by the presence of thymol (44.2%), *E*‐ocimene (25.8%) and *α*‐terpinene (12.3%) as the main chemical constituents. On the other hand, the study carried out by Benjilali et al. [22] on 14 samples of essential oils of *T. ciliatus* collected in different regions of Morocco showed a variation in the percentage and nature of the majority compounds according to the geographical origin of this plant: thymol (0.3%–29.3%), carvacrol (0.4%–21.7%), *α*‐terpenyl acetate (0%–42.9%), geranyl acetate (0%–21.7%), geranyl butyrate (0%–26.7%), camphor (0.4%–28.4%), and borneol (0.1%–31.6%).

On the other hand, many studies showed great diversity in the chemical composition of the essential oils of the species belonging to the genus of Thymus, among these a study carried out by Giordani et al. [15] on the essential oils of *T. numidicus* and *T. algeriensis* from the region of souk Ahras (East of Algeria) where the dominant compounds of the essential oil of *T. numidicus* were thymol (66.31%–57.20%) followed by linalool (8.62%–9.26%), *γ*‐terpinene (6.12%–9.19%) and *p*‐cymene (6.20%–7.55%), while those of *T. algeriensis* were α‐pinene (27.14%–25.52%) followed by camphora (8.77%–8.45%) 1,8‐cineole (7.69%–7.68%), and sabinene (5.25%–5.61%).

Another research performed by Imelouane et al. [23] revealed that the major compounds of the essential oil of *T. vulgaris* from Morocco were camphora (38.54%), camphene (17.19%), α‐pinene (9.35%), 1,8‐cineole (5.44%), borneol (4.91%), and *β*‐pinene (3.90%).

These differences in essential oil composition may be due to several factors such as climatic factors specific to the regions of harvest, geographical factors such as latitude and soil type, harvesting period, chromatographic analysis conditions, extraction technique, and the type of species [24, 25].

### 2.3 Dosage of Total Polyphenols and Flavonoids in Crude Extracts of *T. ciliatus*

The total polyphenol content is calculated using the regression equation of the gallic acid calibration range (*y* = an *x* + b). It is measured in micrograms of gallic acid equivalent per milligram of extract. The total flavonoids are calculated using a linear calibration curve (*y* = an *x*) performed by the standard quercetin. It is measured in milligrams of extract per microgram of quercetin equivalent (Table 3)

**TABLE 3:** Total polyphenol and flavonoid contents of *T. ciliatus* extracts.

	*T. ciliatus extracts*	
	Total polyphenols (µg EAG/mg extract)	Total flavonoids (µg EQ/mg extract)
**Aqueous extract**	286.5 ± 0.62	29.11 ± 1.69
**Methanolic extract**	326.27 ± 2.80	54.68 ± 3.97
**Acetonic extract**	163.11 ± 0.41	24.14 ± 1.14
**Dichloromethane extract**	25.69 ± 3.84	6.25 ± 0.00

The values represent the mean ± SD of three measurements. Means with different superscript letters in the same column are significantly different (*p* < 0.05)., EGA: Equivalent Galic Acid, EQ: Equivalent Quercetin.

Table 3 represents the total phenolic and flavonoid components. The total phenolic content of the extracts varied between 25 693.84 g EAG/mg for the dichloromethane extract and 326.27 g EAG/mg for the methanol extract. Similarly, the flavonoid content of the extracts revealed that the dichloromethane extract had the lowest quantity (6250.00 g EQ/mg extract), while the methanol extract had the highest value (654 683.97 g EQ/mg extract).

The results showed that the different extracts of the aerial part of our plant contained decreasing levels of polyphenols: Methanolic extract>Aqueous extract>Acetone extract>Dichloromethane extract. Methanolic extracts have the best total polyphenol content (326.27 ± 2.80; 146.1324 ± 2.39 µg EAG/mg extract) compared with the other extracts. This could be due to the fact that the methanol solvent solubilizes most of the plant phenolic components. It is also noted that the polyphenol content depends on the type of solvent used as well as the type of plant used. This is confirmed by Falleh et al. [26], who reported that methanol is the appropriate solvent for higher polyphenol recovery.

Moreover, the results show that methanolic extract has the highest flavonoid value (54.68 ± 3.97 µg EQ/mg extract) followed by aqueous extract (29.11 ± 1.69 µg EQ/mg extract), then acetonic extract (24.14 ± 1.14 µg EQ/mg extract) and lastly the dichloromethane extract (6.25 ± 0.00 mg EQ/g extract); it means that the results of the total flavonoids determination are in accordance with those of total polyphenols.

### 2.4 Antioxidant Activity

The antioxidant activity of plant extracts is mainly due to phenolic chemicals. Due to the need to replace synthetic compounds used in the food sector for preservation or nutrition, natural antioxidants are in high demand [27]. In fact, antioxidants have several mechanisms of action, including anti‐radical activity, iron fixing, and hydrogen subtraction chain disruption. The antioxidant impact of a compound‐rich extract must be studied using many methodologies to account for each compound's mode of action [28]. In addition, the results reported in Table 4 reveal that the polar and medium polar extracts of *T. ciliatus* showed the significant scavenging ability of 2,2'‐azino‐bis‐(3‐ethylbenzothiazoline)‐6‐sulfonic (ABTS) radical with half‐maximal inhibitory concentration (IC_50_) values of 16.992.11, 21.230.39, and 21.570.25 g/mL recorded with acetonic, methanolic and aqueous extracts, respectively, and 2,2‐diphenyl‐1‐picrylhydrazyl (DPPH) radical with IC_50_ values of 10.990.59 and 50.620.54 g/mL recorded with acetonic and methanolic extracts, respectively. However, only a low scavenging activity of this radical is noticed with the aqueous extract. These extracts also showed considerable capacity in the reduction of copper and phenantroline ions, and low capacity in the reduction and chelation of iron. Moreover, the apolar extracts of *T. ciliatus*, which are dichloromethane extract and essential oil extract, gave nothing or very low antioxidant capacity with all the tests performed [29]. In contrast, the essential oil had a weak antioxidant activity, possibly due to its content of oxygenated sesquiterpenes [16]. In a recent study by Tefiani et al. [30], the antioxidant activity of *T. ciliatus* from Tlemcen was investigated (hydroxyl radical, ORAC, DPPH, ATBS, TBARS, and H_2_O_2_) exclusively for the essential oil. This essential oil was only useful in hydroxyl radical scavenging. This agrees with our results, where the essential oil's antioxidant impact was found in just three of six experiments and with low IC_50_ values.

**TABLE 4:** Antioxidant activity of the essential oil and crude extracts of *T. ciliatus*.

Essential oil and crude extracts	DPPH IC_50_ (µg/mL)	ABTS IC_50_ (µg/mL)	reducing power A0.5 (µg/mL)	Metal chelate IC_50_ (µg/mL)	CUPRAC A0.5 (µg/mL)	Phenanthroline A0.5 (µg/mL)
**Aqueous**	123.10 ± 5.15	21.57 ± 0.25	>200	90.81 ± 1.57	56.40 ± 1.80	163.09 ± 2.70
**Methanolic**	10.99 ± 0.59	21.23 ± 0.39	161.66 ± 27.27	351.08 ± 8.67	8.69 ± 3.50	89.36 ± 1.27
**Acetonic**	50.62 ± 0.54	16.99 ± 2.11	133.43 ± 3.08	>800	41.58 ± 1.66	170.57 ± 0.47
**Dichloromethane**	>800	808.21 ± 16.56	>200	>800	181.74 ± 11.06	>800
**Essential oil**	>800	303.71 ± 7.63	>200	145.04 ± 1.23	464.83 ± 3.69	>800
**Standard**						
**BHA**	6.14 ± 0.41	1.81 ± 0.10	9.29 ± 0.22	NT	5.35 ± 0.71	NT
**BHT**	12.99 ± 0.41	1.29 ± 0.30	NT	NT	8.97 ± 3.94	5.28 ± 0.34
**EDTA**	NT	NT	NT	8.57 ± 0.14	NT	NT

All values represent the mean ± SD of three measurements. Means with different superscript letters in the same column are significantly different (*p* < 0.05).

### 2.5 Antimicrobial Activity

Antimicrobial activity was tested using the agar diffusion and disc methods. These methods are inspired by the antibiogram method; the antimicrobial activity is thus expressed by measuring the diameter of the zone of inhibition around a disk or a well containing the extracts to be tested. The results obtained are shown in Table 5.

**TABLE 5:** Diameter of the zones of inhibition of bacterial growth induced by the different extracts of *T. Ciliatus*.

Microbial strains	Diameter of the inhibition zone in mm											
	Aqueous extract		Methanolic extract		Acetonic extract		Dichloromethane extract		Essential oil		DMSO	GN/NY
	Disk	Well	Disk	Well	Disk	Well	Disk	Well	Disk	well		
*E. coli* ATCC 25922	NA	NA	NA	NA	NA	NA	NA	NA	**10.00 ±** **0.00**	**9.00 ±** **0.00**	NA	20
*P. aeruginosa* ATCC 27853	10.00 ± 0.00	NA	10.00 ± 1.00	NA	9.00 ± 0.50	NA	8.00 ± 1.00	NA	**10.00 ± 0.25**	**NA**	NA	17
*B. cereus* ATCC 10876	NA	NA	13.00 ± 1.00	10.00 ± 0.50	10.00 ± 1.00	8.00 ± 0.50	9.00 ± 1.00	10.00 ± 0.00	**12.33 ± 0.58**	**12.00 ±** **0.00**	NA	30
*S. aureus* ATCC 25923	NA	NA	7.00 ± 0.00	10.00 ± 0.00	NA	NA	NA	12.00 ± 1.00	**12.00 ±** **1.00**	**19.5 ±** **0.57**	NA	33
*C. albicans* ATCC 10231	9.00 ± 0.00	NA	10.00 ± 0.00	12.00 ± 0.00	11.50 ± 0.70	10.00 ± 0.00	10.00 ± 0.00	11.00 ± 0.25	**14.5 ±** **0.70**	**12.00 ±** **0.00**	NA	36

Values are Mean ± SD of three measurements. Values with different superscript letters in the same column are significantly different (Tukey test, *p* ≤ 0.05).

According to the results in Table 5, all crude extracts showed significant inhibitory efficacy against all target bacteria except *Escherichia coli*. The most active extract is the methanolic extract.

The essential oils showed significant antimicrobial activity against all the tested strains, with particularly high efficacy against Gram‐positive bacteria such as *S. aureus* and *B. cereus*, as well as against the yeast *C. albicans*, with inhibition zones of **Ø = 12.00 and 19.50 mm**, **Ø = 12.00 and 12.33 mm**, and **Ø = 12.00 and 14.5 mm**, respectively. However, weaker activity was observed against Gram‐negative bacteria, *E. coli* (**Ø = 9.00 and 10.00 mm)**, and *P. aeruginosa*  **(Ø = 6.00 and 10.00 mm)**. This suggests that the essential oils are more active against Gram‐positive bacteria than against Gram‐negative bacteria, which may be due to the hydrophilic nature of the cell walls of Gram‐negative bacteria, preventing the penetration of the hydrophobic components of the oils. Gram‐positive bacteria are therefore more sensitive to essential oils [34]. The differences observed may also be related to the chemical composition of the extracts and essential oils, where the antimicrobial activity is mainly attributed to the major compounds. In comparison with previous studies, essential oils from Thymus species are highly active, due to their richness in carvacrol and thymol, phenolic compounds known for their strong antimicrobial properties [16, 31]. However, the essential oils of our plant contain primarily compounds such as terpinenyl acetate, camphor, caryophyllene oxide, trans‐nerolidyl acetate, 1,8‐cineole, epiglobulol, bornyl acetate, and borneol, which, while effective, do not show as strong antimicrobial activity as thymol and carvacrol. Regarding bacterial reactivity, the results showed that *B. cereus* was the most sensitive strain, developing resistance only to the aqueous extract, but remaining less sensitive compared to gentamicin (GM) (inhibition zone of 30 mm). In contrast, the *E. coli* strain showed strong resistance to all tested extracts except the essential oils, which is not surprising given the literature reporting the resistance of this species to antibiotic agents. Overall, the inhibition zones obtained with our extracts were smaller than those obtained with the reference antimicrobial, which can be explained by the higher concentration of the antimicrobial on the disc, its degree of purity, and its toxicity.

Furthermore, the antimicrobial activity of the essential oil and the various crude extracts was evaluated by estimating the minimum inhibitory concentration (MIC) and the minimum bactericidal concentration (MBC) to determine the levels of effectiveness of these extracts against the various targeted strains (Table 6).

**TABLE 6:** Minimum inhibitory concentration (MIC) and minimum bactericidal concentration (MBC) of antimicrobial activity of *T*. *ciliatus* extracts.

Microbial strains	MIC (mg/mL)					MBC (mg/mL)			
	H_2_O‐E	ME‐E	ACT‐E	DCM‐E	EOs	H_2_O‐E	ME‐E	ACT‐E	DCM‐E
*E. coli* ATCC 25922	NA	NA	NA	NA	C/4	NA	NA	NA	NA
*P. aeruginosa* ATCC 27853	C/2	C/16	C/2	C/2	C/16	NA	NA	NA	NA
*B. cereus* ATCC 10876	NA	C/2	C/2	C/2	C/8	NA	NA	NA	NA
*S. aureus* ATCC 25923	NA	C	NA	NA	C/4	NA	NA	NA	NA
*C. albicans* ATCC 10231	C/4	C/4	C/8	C/4	C/4	NA	NA	NA	NA

According to the results reported in Table 6, the MIC values were recorded with all the tested extracts and substantially with the essential oils at different dilutions, of which the bacterium *P. aeruginosa* represents the most sensitive germ with a MIC value of C/16 mg/mL. On the other hand, the MBC values were recorded only with the essential oils.

The results of the inhibitory activity of the extracts (aqueous, methanol, acetone, and dichloromethane) and essential oils of *T. ciliatus* against the fungus *Fusarium oxysporum* are summarized in Table 7.

**TABLE 7:** Results of the inhibitory activity of *T. ciliatus* extracts against the fungus *Fusarium oxysporum*.

	Aqueous extract	Methanolic extract	Acetonic extract	Dichloromethane extract	EOs
**% of inhibition**	46.26%	49.25%	38.80%	23.88%	67.16%

The data reveal that the inhibitory activity against the fungus *F. oxysporum* is more interesting with the essential oils than with the crude extracts, with an inhibition rate of 67.16%. Among the crude extracts, the methanolic extract is the most effective, with an inhibition rate of 49.25%, followed by the aqueous extract with a percentage of 46.26%, and finally, the acetone extract and the dichloromethane extract, with a percentage of 38.80% and 23.88%, respectively. The antimicrobial activity is primarily attributed to the presence of mono‐ and sesquiterpenes containing aromatic rings and phenolic hydroxyl groups, which are capable of forming hydrogen bonds with the active sites of the target [31]. The mechanisms through which essential oils inhibit microorganisms involve various modes of action, often linked to their hydrophobic nature. As a result, they integrate into the lipid bilayer of the cell membrane, increasing its permeability, which causes leakage of essential cellular contents. Additionally, the depletion of bacterial enzyme systems may also contribute to this antimicrobial effect [32]. Similarly, a number of effects have been reported for fungi including inhibition of sporulation, germination, hyphal elongation, and disruption of cell walls and membranes [33]. To our knowledge and according to the available literature, no antifungal activity study against the tomato rot fungus *F. oxysporum* has been conducted with extracts of the plant *T. ciliatus*. Only one previous study by El Ajjouri et al. [34], demonstrated that the essential oil of *T. ciliatus* has a strong inhibitory activity against four wood rot fungi (*Gloeophyllum trabeum*, *Poria placenta*, *Coniophora puteana*, and *Coriolus versicolor*). Furthermore, Nadia et al. [35] reported that the essential oils of *T. vulgaris* have antifungal power in particular against the fungus *F. oxysporum*.

### 2.6 Anti‐diabetic Activity

The different extracts were also examined in vitro for their inhibitory activity toward the enzyme α‐glucosidase, using the colorimetric method in 96‐well microplates.

The obtained results showed that the high inhibition capacity of α‐glucosidase was noticed with the aqueous extract and recorded an IC_50_ in the order of 2.560 g/mL, followed by the essential oils with an IC_50_ value of 57.114 g/mL. The extracts of methanol, acetone, and dichloromethane showed a low capacity of inhibition of the α‐glucosidase enzyme compared to the standard Acarbose, whose lowest inhibitory effect is recorded with the methanolic extract with an IC_50_ in the order of 718.624 g/mL (Table 8).

**TABLE 8:** Effect of essential oil and crude extracts of *T. ciliatus* vis‐a‐vis the enzyme α‐glucosidase.

% of inhibition								
**Extracts**	**15.625 µg/mL**	**31.25 µg/mL**	**62.5 µg/mL**	**125 µg/mL**	**250 µg/mL**	**500 µg/mL**	**1000 µg/mL**	**IC_50_ (µg/mL)**
**H_2_O‐E**	50.00 ± 0.00	83.28 ± 3.04	86.47 ± 1.89	89.25 ± 0.63	90.71 ± 0.83	91.82 ± 1.90	93.06 ± 2.28	2.56 ± 0.06
**MeOH‐E**	12.03 ± 2.72	18.64 ± 0.86	21.31 ± 1.94	27.95 ± 1.43	28.67 ± 1.68	37.25 ± 3.05	54.18 ± 2.72	718.62 ± 4.13
**ACT‐E**	6.69 ± 1.40	9.46 ± 0.45	16.37 ± 4.95	20.06 ± 0.43	36.41 ± 0.39	47.92 ± 1.92	66.15 ± 3.64	452.19 ± 0.40
**DCM‐E**	6.69 ± 1.40	11.21 ± 1.41	18.94 ± 5.06	20.04 ± 4.51	37.12 ± 1.65	48.09 ± 0.00	62.94 ± 0.08	493.54 ± 0.81
**EOs**	38.77 ± 6.08	47.52 ± 0.64	50.38 ± 2.91	54.69 ± 1.26	58.01 ± 2.49	74.07 ± 1.36	86.24 ± 0.81	57.11 ± 4.39
**Acarbose**	27.43 ± 2.18	38.91 ± 3.20	54.86 ± 1.79	67.29 ± 2.63	80.19 ± 1.66	85.54 ± 0.45	91.05 ± 0.72	275.43 ± 1.59

Values are presented as means ± SD of three measurements. Values with different superscript letters in the same column are significantly different (Tukey test, *p* ≤ 0.05).

The inhibitory effect of α‐glucosidase is also determined as an important strategy to manage type II diabetes. The essential oil and aqueous extract were the main inhibitors of α‐glucosidase. This action can be attributed to the presence of these extracts of compounds with a competitive inhibition capacity of α‐glucosidase, preventing postprandial hyperglycemia. In fact, several natural molecules, including terpenes, alkaloids, flavonoids, phenylpropanoids, quinines, and others, have been isolated and reported as inhibitors of α‐glucosidase [36]. In a study conducted by Tefiani [30], it was found that the essential oil extracted from *T. ciliatus* ssp. *eucilatus* from Tlemcen exerted a good antidiabetic activity (anti‐alpha amylase) (5.198 µg EA.g^.k^ EO). In addition, previous works have described the antidiabetic activity of other extracts of species of the genus *Thymus*, including a study by Hyun et al. [37], which demonstrated that the methanolic extract of the species *Thymus quinquecostatus* exerted a significant antidiabetic activity recording an IC_50_ value of 4.39 µg/mL. In general, plants have different active principles that allow them to have an action on the organism. In the case of diabetes, they have a hypoglycemic action, whose mechanism differs as well as the active principle responsible. Plant constituents with hypoglycemic activity include polysaccharides, peptides, alkaloids, glycopeptides, triterpenoids, amino acids, steroids, flavonoids, phenols, coumarins, inorganic ions, and guanidines [38]. In this context, the antidiabetic activity of several extracts of numerous plants has been extensively studied [39].

### 2.7 Anti‐BChE Activity

The results of the anti‐BChE activity are expressed as IC_50_. Galantamine is used as a reference substance. According to Table 9, our results show a very significant ability of all *T. ciliatus* extracts as inhibitors of the BChE enzyme. Butylcholinesterase inhibition with an IC_50_ of 0.260 µg/mL, followed by the EO extract with an IC_50_ of 2.960 µg/mL, then the acetone extract, the methanolic extract, and finally the aqueous extract (6.690, 14.230, and 19.820 µg/mL), respectively. This means that the anti‐BChE activity of our extracts is proportional to the polarity of the solvents used. These results allowed us to classify the extracts as follows: dichloromethane extract > essential oil extract > acetone extract > methanolic extract > aqueous extract. The effects of all these extracts were more effective than the standard (galantamine, IC_50_ = 34.75 µg/mL).

**TABLE 9:** Anti‐BChE activity of EO and extracts.

EO and crude extracts	Butyrylcholinesterase inhibitory activity							
	6.25 µg	12.5 µg	25 µg	50 µg	100 µg	200 µg	400 µg	IC_50_ (µg/mL)
**Aqueous**	44.18 ± 2.25	49.49 ± 2.24	51.96 ± 1.44	54.04 ± 1.98	54.80 ± 0.30	56.02 ± 0.80	65.45 ± 3.84	19.82 ± 0.45
**Methanolic**	43.50 ± 4.38	51.13 ± 0.97	54.04 ± 3.17	58.12 ± 1.92	60.12 ± 2.88	61.34 ± 3.23	74.13 ± 4.26	14.23 ± 0.40
**Acetonic**	48.20 ± 0.37	56.02 ± 4.19	68.41 ± 1.40	72.04 ± 1.76	75.04 ± 2.08	80.18 ± 2.13	90.17 ± 3.07	6.69 ± 0.62
**Dichloromethane**	50.60 ± 0.57	74.84 ± 2.65	81.29 ± 3.74	82.98 ± 0.,12	83.25 ± 0.91	93.16 ± 3.91	96.60 ± 4.10	0.26 ± 0.20
**Essential Oil**	50.33 ± 0.16	64.22 ± 0.25	69.92 ± 2.27	73.12 ± 3.33	76.79 ± 2.96	85.81 ± 2.77	92.84 ± 0.92	2.96 ± 0.80
**Galantamine**	3.26 ± 0.62	6.93 ± 0.62	24.03 ± 2.94	45.13 ± 2.60	63.87 ± 2.85	73.57 ± 0.77	78.95 ± 0.58	34.75 ± 1.99

Values are presented as means ± SD of three measurements. Values with different superscript letters in the same column are significantly different (Tukey test, *p* ≤ 0.05).

BChE has been identified as an important drug target for the diagnosis and treatment of Alzheimer's disease. This enzyme is involved in the degradation of the neurotransmitter in neuronal synapses after its action on postsynaptic receptors. Therefore, inhibiting the action of BchE on the neurotransmitter can increase its concentration and improve the cognitive abilities of individuals with Alzheimer's disease. Several natural compounds have been reported to inhibit BChE, with alkaloids being the most potent compounds, followed by terpenes [40]. According to the research, the analyzed extracts had significant anti‐BChE action. Furthermore, the high anti‐BChE action of the dichloromethane extract could be attributed to the presence of terpene chemicals, since alkaloids have not been reported in this species. On the other hand, the essential oil had a less strong effect than the dichloromethane extract, which could be explained by the terpene content of the EO. In addition, according to the literature, most of the known inhibitors of AChE and BChE enzymes are alkaloids and terpenes [40]. Currently, many studies have been conducted to identify other natural compounds that could have significant anticholinesterase activity. Among them, the study of Houghton et al. [41], demonstrated that compounds other than alkaloids and terpenes exhibited a great ability to inhibit cholinesterase enzymes such as phenolic compounds, flavonoids, and coumarins. Therefore, our results further confirm the role of polyphenols and terpenes in anticholinesterase activity.

## 3 Conclusions

In the present study, the chemical characterization and pharmacological effects of crude extracts and the essential oil of *T. ciliatus* growing in Algeria were investigated. The results showed a significant potential of the plant to inhibit BChE, which is an important target for the treatment of Alzheimer's disease. On the other hand, the essential oil of *T. ciliatus* showed good antimicrobial activity against all the targeted strains. In addition, the extracts showed a remarkable inhibitory effect on α‐glucosidase. Based on these results, *T. ciliatus* can be considered an important source of bioactive compounds with anti‐Alzheimer's, anti‐microbial, anti‐diabetic, and antioxidant properties.

## 4 Experimental

### 4.1 Extraction and GC‐MS Characterization of *T. ciliatus* Essential Oil

The species *T. ciliatus* was collected during the flowering period, on March 15, 2020, in the region of Djebel Taraf, Area 2251 ha, altitude 1134m (wilaya of Oum El‐Bouaghi—Algeria), then identified by taxonomic experts (Pr. Rebbas Khalef of the University of M'sila—Algeria). The sample was preserved, and the voucher of the specimen, coded P202086, was deposited in the herbarium of the Department of Biology of the Faculty of Sciences at the University of Oum El‐Bouaghi, Algeria, for future reference. The essential oil of *T. ciliatus* was extracted by hydrodistillation [42]. The chemical characterization of *T. ciliatus* essential oil was carried out at the plant research laboratory of the Department of Chemistry, Tokat Gaziosmanpasa University, Turkey, by gas chromatography technique under the following analytical conditions:

- The carrier gas used is Helium whose flow rate was set at 1 mL/min.
- The temperature of the injector ass 250°C.
- Injection in split mode (1/100) of 10 µL of essential oil.
- The temperature of the detector was 250°C.
- The oven temperature was maintained at 50°C for 10 min, then gradually increased to 260°C at a rate of 5°C/min.

The retention indices of all compounds were determined following the method of Van den Dool and Kratz, using n‐alkanes as reference standards. Compound identification was carried out by comparing their mass spectra with the Wiley and NBS databases, as well as the descriptions provided by Adams, and by cross‐referencing their retention indices with published data.

### 4.2 Preparation of Crude Extracts of *T. ciliatus*

The preparation of the crude extracts was carried out by maceration; 20 g of the dry plant material of the aerial part was put in contact with 200 mL of the selected solvent (methanol [70%], water, acetone, and dichloromethane) for 3 days at room temperature and protected from the light. After filtration under vacuum, the filtrates were concentrated by evaporation to obtain organic extracts and by freeze‐drying to obtain aqueous extracts.

### 4.3 Determination of Total Polyphenols

The total polyphenol content in the extracts was quantified using the Folin‐Ciocalteu colorimetric method, which involves a mixture of phosphotungstic and phosphomolybdic acids [43]. A 20 µL aliquot of the extract was combined with 1 mL of freshly prepared Folin‐Ciocalteu reagent (diluted tenfold) and 75 µL of 7.5% sodium carbonate (Na₂CO₃). The mixture was incubated at room temperature for 30 min, and absorbance was measured at 765 nm against a blank using a spectrophotometer. Results were expressed as milligrams of gallic acid equivalents per gram of dry plant material.

### 4.4 Dosage of Total Flavonoids

The total flavonoid content in our extracts was quantified using the colorimetric method with aluminum chloride (AlCl₃). Aluminum chloride reacts with flavonoids in the extracts to form a yellow complex, which absorbs at 510 nm in the visible spectrum. The coloration produced is proportional to the total flavonoid content in the extracts. A 50 µL amount of the crude extracts were mixed with 130 µL of MeOH and subsequently with 10 µL (CH_3_COOK). After 5 min, 10 µL (of a 10% AlCl_3_ solution was added. The whole is stirred with a vortex and incubated for 40 min. Reading of the absorbance at 415 nm against a blank (the blank is prepared by replacing the reagents with methanol (50 µL extract + 150 µL methanol).

### 4.5 Antioxidant Activity

#### 4.5.1 DPPH Radical Scavenging Activity

The antioxidant activity of plant extracts was assessed using the DPPH free radical method, as described by Blois [44]. In a 96‐well microplate, 40 µL of each extract at varying concentrations was mixed with 160 µL of a 1 mM DPPH solution in methanol. The mixture was incubated at room temperature in the dark for 30 min. Absorbance was then measured at 517 nm using a microplate reader (Perkin Elmer, Enspire). Butylated hydroxyanisole (BHA) and butylated hydroxytoluene (BHT) were used as standards. The inhibition percentage was calculated using the formula (1):

(1) $$I\;\left(\%\right)=\left[\left(\mathrm{Abs}\;\mathrm{control}\;-\;\mathrm{Abs}\;\mathrm{test}\right)/\mathrm{Abs}\;\mathrm{control}\right]\;\times \;100$$

where I (%) is the percentage of inhibition, and Abs are the absorbencies of the control and the test sample after 30 min respectively. IC_50_ values corresponding to the extract concentration inducing a reduction of the absorbance of the DPPH solution by 50% were determined from the inhibition curve.

#### 4.5.2 ABTS Scavenging Capacity

ABTS radical scavenging activity was determined according to the method described by Re et al. [45]. ABTS^·+^ solution was prepared by mixing 7 mM of ABTS·^+^ and 2.45 mM of potassium persulfate (K_2_S_2_O_8_) for 16 h. Before using the absorbance of the solution was adjusted with distilled water to an absorbance of 0.7 ± 0.025 at 734 nm. The reactionary medium consisted of 160 µL of ABTS^·+^solution and 40 µL of extract at different concentrations in a 96‐well microplate. The absorbance was read at 734 nm after 10 min incubation and the percentage of inhibition was calculated by the previous formula (1). The results were compared to BHA and BHT used as standards.

#### 4.5.3 Reducing Power

The reduction of iron ions (Fe^3^⁺) was assessed using the reducing power method of Oyaizu [46], with some modifications. A 10 µL aliquot of each extract at varying concentrations was mixed with 40 µL of phosphate buffer (0.2 M, pH = 6.6) and 50 µL of a 1% potassium ferricyanide (K₃Fe(CN)₆) solution. The mixture was then incubated at 50°C for 20 min. After incubation, 50 µL of 10% trichloroacetic acid, 40 µL of distilled water, and 10 µL of a 0.1% ferric chloride (FeCl₃) solution were added to each well. Absorbance was recorded at 700 nm using a microplate reader (Perkin Elmer, Enspire, Singapore). BHA and BHT served as positive controls. The concentration corresponding to an absorbance of 0.5 (denoted as A_0.5_) was determined for each extract from the curve of absorbance versus concentration.

#### 4.5.4 Iron Chelation

The chelation activity of iron ions by extracts was determined using the Ferene method Deckeret Welch [47]. A volume of 40 µL of plant extract at different concentrations was mixed in a 96‐well microplate with 40 µL of methanol, 40 µL of the iron chloride solution (FeCl_3_, 0.2 mM), and 80 µL of Ferene (0.5 mM). The mixture was incubated for 10 min and the absorbance was read at 593 nm. The chelation activity of the extracts was compared to the standard Ethylene diamine tetraacetic acid (EDTA). Results are expressed as the percentage of inhibition determined by the previous formula (1).

#### 4.5.5 Cupric Ion Reducing Antioxidant Capacity

The ability of the extracts to reduce copper ions (Cu^2^⁺) was evaluated using the cupric reducing antioxidant capacity (CUPRAC) method [48]. A 40 µL aliquot of each plant extract at varying concentrations was added to a 96‐well microplate. Then, 50 µL of CuCl₂ (10 mM), 50 µL of neocuproine (7.5 mM), and 60 µL of CH₃COONH₄ buffer (1 M, pH = 7.0) were added to each well. The microplate was incubated in the dark for 1 hour, and the absorbance was measured at 450 nm using a microplate reader. A_0_._5_ values were determined from the absorbance curve. BHA and BHT were used as standards.

#### 4.5.6 Phenanthroline Method

The antioxidant activity of our extracts was assessed using the phenanthroline method, as described by Szydlowska‐Czerniak (2008) [49]. A 10 µL aliquot of the plant extract was added to a 96‐well microplate, followed by 50 µL of FeCl₃ (0.2%) and 30 µL of phenanthroline (0.5%). Then, 110 µL of methanol (MeOH) was added, and the mixture was incubated in the dark at 30°C for 20 min. The absorbance was measured at 510 nm, with BHT used as the standard. Results were expressed as A₀.₅, the concentration corresponding to an absorbance of 0.50 (µg/mL).

### 4.6 Antimicrobial Activity

#### 4.6.1 Targeted Strains

Microbial strains used in this experiment were 4 selected bacterial strains pathogenic and/or involved in the food spoilage process. Two Gram (−) bacteria: *E. coli* ATCC 25922; *P. aeruginosa* ATCC 27853 and two Gram (+) bacteria: *Bacillus cereus* ATCC 10876; *S. aureus* ATCC 25923.

Two fungal strains: *C. albicans* ATCC 10231 and *F. oxysporum* f.sp *lycopersici*, microorganisms were obtained from the culture of the laboratory collection of microbiology of the Center of Research in BioTechnology of Constantine—Algeria.

#### 4.6.2 The Disc Method

This method was used to evaluate the sensitivity of the tested strains to the studied extracts according to the method described by Berghe and Vlietinck [50] with some modifications. Within 15 min of adjusting the density of the bacterial or fungal suspension (the inoculum), a swab was dipped into the suspension and the entire surface of the agar (Mueller Hinton for bacteria or Sabouraud for yeast) was swabbed in three replicates by rotating the plate each time with 60°, and finally, swabbed all around the edge of the agar surface. Under aseptic conditions and using sterile forceps, 6‐mm diameter disks of sterile Wattman number 4 paper (sterilized at 120°C for 15 min by autoclaving) are placed on the agar previously inoculated with the chosen strain, then soaked with 10 µL of the different extracts to be tested with a concentration of 8 mg/mL.

Dimethyl sulfoxide (DMSO)‐impregnated discs are used as negative controls. GM (= 10 mg) is used as a positive control for bacterial strains, while Nystatin is used as a positive control for yeast. Petri dishes are then incubated in an oven at 37°C for 24 h for bacteria and 30°C for 48 h for yeast. Antimicrobial activity is determined by measuring the diameter of the zone of inhibition around each disc using a caliper (including the disc diameter, 6 mm). The test is performed in triplicate to minimize experimental errors (CLSI‐M2‐A9 2006).

#### 4.6.3 The Well Method

The principle and procedure of this method are the same as the previous method but discs are substituted with wells. First, Petri dishes cast with Muller Hinton agar were inoculated by flooding using a volume of 1000 µL of the microbial suspension (106 CFU/mL). Then, 6‐mm diameter and 4‐mm deep wells were made in the agar using the sterile Pasteur pipette. Then, a 50 µL volume of the test extract (8 mg/mL) diluted in DMSO was placed in one well (one extract for each well). One well containing DMSO was used as a negative control (CLSI‐M7‐A7,2006). Finally, the Petri dishes were left at room temperature for 15 min to allow the volume to diffuse into the agar and then incubated in an oven with different temperatures and incubation times depending on the target microorganism. Antimicrobial activity was assessed by measuring the inhibition diameter in millimeters. The test was performed three times and the values are the averages of three replicates.

#### 4.6.4 Determination of MIC and MBC

For MIC determination, the method consists of adding 50 µl of the microbial suspension in each microplate well containing 50 µL of each dilution of each extract to be tested. The microplate is then incubated in an oven at 37°C for 24 h. The MIC is defined as the lowest concentration of the extract for which the microorganism does not show visible growth. For the determination of the MBC, the method consists in placing spots from the MBC positive wells in petri dishes containing Muller Hinton agar. The plates are then incubated at 37°C for 24 h. The MBC is defined as the lowest concentration of the extract that results in the death of the microorganism of interest after 24 h of incubation [51].

#### 4.6.5 Direct Contact Method

This method is used to evaluate the antimicrobial power of our extracts against the phytopathogenic fungus *F. oxysporum*. First, Petri dishes are aseptically poured with the Potato Dextrose Agar (PDA) culture medium and the extract is to be tested (5 mg of extract in 1 mL of DMSO). Then, a 5 mm diameter disc taken from a young mushroom culture is aseptically placed in the center of the petri dish containing the PDA and the extract to be tested. Finally, the Petri dishes are incubated in an oven at 28°C for 7 days. After incubation, the mycelial growth of the fungus is measured on a millimeter scale using a ruler. The control is prepared by replacing the extract with DMSO, and the experiment is repeated 4 times for each extract [52].

The inhibitory activity is expressed as a percentage and calculated according to the following formula (2):

(2) $$I=\left(C-T/C\right)\times 100$$

I = inhibition rate in %.

C = Radial growth of the fungus in mm on the PDA with DMSO (control)

T = Radial growth of the fungus in mm on the PDA containing the extract.

#### 4.6.6 Anti‐diabetic Activity

The antidiabetic activity of our extracts at various concentrations was evaluated against the α‐glucosidase enzyme, following the method of Kee et al. [53] with some modifications. In this experiment, 50 µL of plant extract at different concentrations and 50 µL of p‐nitrophenyl‐α‐D‐glucopyranoside substrate solution were added to a microplate. The mixture was then incubated at 37°C for 10 min. Following incubation, 100 µL of α‐glucosidase enzyme solution was added. Absorbance was measured at 405 nm using a microplate reader. The α‐glucosidase inhibitory activity was expressed as a percentage of inhibition, and IC50 values were calculated. Acarbose was used as a positive control.

#### 4.6.7 Inhibitory Activity of BChE

The inhibitory effect against BChE was assessed using the spectrophotometric Ellman method [54]. In a 96‐well microplate, 150 µL of sodium phosphate buffer (0.1 M, pH = 8) was mixed with 10 µL of plant extract at varying concentrations and 20 µL of BChE enzyme (6.85 × 10⁻^3^ U). The microplate was incubated at 25°C for 15 min, after which 10 µL of DTNB solution [5,5‐dithio‐bis (2‐nitrobenzoic acid)] (0.5 mM) and 20 µL of acetylthiocholine iodide (0.71 mM) or butyrylthiocholine chloride (0.2 mM) were added. Absorbance was recorded after 15 min at 412 nm. Galantamine was used as the positive control.

#### 4.6.8 Statistical Analysis

The results are presented as mean ± standard deviation (SD). One‐way analysis of variance followed by Tukey's multiple comparisons test was performed using GraphPad Prism version 5. Differences between means were considered statistically significant at *p* < 0.05.

## Author Contributions

**Nadia Djermane**: writing original draft, investigation, formal analysis. **Mostapha Brahmi**: writing original draft, investigation, formal analysis. **Djallal Eddine Adli**: supervision, writing – review and editing, data curation. **Rebbas Khellaf**: supervision, writing – review and editing, data curation. **Stefania Garzoli**: supervision, writing – review and editing, data curation. **Rabah Arhab**: supervision. **Ramazan Erenler**: supervision. **Micaela Gliozzi**: conceptualization, writing – review and editing. **Vincenzo Musolino**: conceptualization, writing – review and editing. All listed authors have made significant, direct, and intellectual contributions to the work and have approved it for publication. They have all read and agreed to the final version of the manuscript.

## Conflicts of Interest

The authors declare no conflicts of interest.

## Data Availability

The data will be available on request to the authors.
